# Oral Health Inequalities and the Corporate Determinants of Health: A Commentary

**DOI:** 10.3390/ijerph17186529

**Published:** 2020-09-08

**Authors:** Lisa Jamieson, Barry Gibson, W. Murray Thomson

**Affiliations:** 1 Australian Research Centre for Population Oral Health, University of Adelaide, Adelaide SA 5005, Australia; 2 School of Dentistry, University of Sheffield, Broomhall, Sheffield S10 2TG, UK; b.j.gibson@sheffield.ac.uk; 3 School of Dentistry, University of Otago, North Dunedin, Dunedin 9016, New Zealand; murray.thomson@otago.ac.nz

**Keywords:** corporate determinants of health, international oral health inequalities, Lukes “three faces of power” theory

## Abstract

Empirical research critically examining the role of the corporate determinants of health has gained traction in the past few years. Many of these reports have received strong, sometimes litigious, backlash from the corporations exposed. The aim of this paper is to provide a critical commentary on existing literature, policies, procedures and observations of issues, especially regarding the use of the corporate determinants of health as a research construct, in the persistence and flourishing of oral health inequalities at a global level. We discuss theoretical frameworks that underpin the power constructs of the corporate determinants of health, including Lukes “three faces of power” theory. This theory posits that power is exercised in three ways: through decision-making, through non-decision-making and ideologically. We will demonstrate, using examples of corporate determinants of health and oral health inequalities from several countries, how intervening at key leverage points is a crucial strategy for improving oral health inequalities at a global level.

## 1. Introduction

The corporate determinants of health play a substantial role in the persistence and, in many cases, flourishing of oral health inequalities at a global level. There is evidence that the sugar, alcohol and tobacco industries are influencing professional organisations, research and policy through comprehensive and non-transparent corporate strategies. Hastings argued that tackling corporate power should be a public health priority [1]. We endorse these sentiments, particularly in relation to reducing global oral health inequalities. The aim of this commentary is to provide a critical overview on existing literature, policies, procedures and observations of issues, especially regarding the use of the corporate determinants of health as a research construct, in the persistence and flourishing of oral health inequalities at a global level. Using examples of corporate determinants of health and oral health inequalities from several countries, we demonstrate how intervening at key leverage points, acting simultaneously on multiple subsystems, and counteracting über corporate determinants are crucial strategies for improving oral health inequalities at a global level.

## 2. What Are Corporate Determinants of Health?

Health, including oral health, is not only determined by biological and genetic factors but also by the socioeconomic context in which people live their lives [2]. Corporate activity—such as marketing of harmful goods including unhealthy foods, tobacco, sugar-sweetened beverages and alcohol—also affects health [3]. Corporate activities shape our environments and determine the availability, promotion and pricing of consumables [4]. West and Martinue defined the corporate determinants of health as “the factors that influence health which stem from the profit motive” [5]. Kickbusch et al. extended this definition as being the “strategies and approaches used by the private sector to promote products and choices that are detrimental to health” [4]. This singular concept draws together a number of domains. At the individual level, these include consumer and health-related behavior, individualization and choice. At the macro-level, the domains include the global consumer society and the political economy of globalization. Criticisms of the corporate determinants of health can be found in both the health [6,7] and political science [8,9] literature, dating back to the 1920s although not necessarily using “corporate determinants of health” nomenclature.

In 2016, Kickbusch et al. identified the four processes through which transnational corporations exerted influence as marketing, lobbying, corporate social responsibility strategies to “whitewash tarnished reputations”, and extended supply chains [4]. Supply chains are systems of organization, people, activities, information and resources involved in supplying a product or service to a consumer; the more powerful a transnational corporation, the more control they have of the complex and dynamic supply and demand network at an international level. It is important to note that multi-national corporate industries directly target vulnerable population groups, including ethnic minorities, young children and low-income groups, and have aggressive marketing and expansion agendas (particularly the tobacco, sugar and alcohol industries) in many low- and middle-income countries. Through critical examination of the actions of the tobacco, alcohol and food industries, particularly their impacts on health, McKee and Stuckler concluded that power and profit lie at the heart of their complex modus operandi [10]. An effective response to the corporate determinants of health must therefore acknowledge, name and address the power imbalance between global corporations, who are usually accountable only to their owners and shareholders, and the general population through governments [4].

## 3. What Is Power?

Power is a complicated concept. In crude terms, it has been defined as “A getting B to do something that B would not otherwise do” [11]. Lukes later described a theoretical construct entitled the “three faces of power” theory, which posits that power is exercised in three ways: decision-making, non-decision-making and ideologically [12]. Decision-making (visible) power is the most overt, and includes policy preferences revealed through political action, laws and regulation [13]. Non-decision-making (hidden) power includes having access to key decision-makers that is not otherwise available to others; this can, for example, facilitate agenda-setting in debates and make certain issues unacceptable for discussion in “legitimate” public forums [14]. Ideological (invisible) power includes the ability of a group to influence people’s wishes and thoughts, even making them want things that contradict their own self-interest [15]. For the lay public to fully appreciate the corporate determinants of health and their influence on public policy, greater understanding is required of who the players are and what goes on behind the scenes of deliberations in legislatures and in public consultations [16].

McKee and Stuckler defined power in the context of the corporate determinants of health as the ability to (1) define the dominant narrative; (2) set the rules and procedures by which society is governed; (3) determine the living and working conditions, and rights, of ordinary people; and (4) take ownership of knowledge and ideas [10]. They argued that corporations are able to frame dominant narratives on the determinants of health (invisible power). They have agency to create doubt about issues even when there is scientific consensus. Corporations may also influence health determinants through marketing activities, determining what is available in stores and at what price [17]. Through marketing campaigns, corporations also have the ability to influence social norms that, in turn, influence how people work, live and socialise (consider the social acceptance of alcohol use, for example). This is particularly pervasive for socially disadvantaged people, who may be framed as being “welfare cheats”, “other” and “free to be foolish” [18]. The perpetuation of the “othering” of such groups and the victim-blaming narrative in the public discourse serves the interests of those corporations and the politicians whom they fund (that is, have purchased) through political donations, whether overt or covert [19].

As governments have increasingly enforced regulations, corporations have invented new ways to influence how and where decisions are made and to create mechanisms that ensure that they will survive and prosper. For example, they situate their research expertise to define global standards, as demonstrated by the tobacco industry in the 1970s [20]. Corporations seek to influence regulatory bodies and health research associations by placing their advisors on committees and/or boards [21,22]. They additionally prefer secret tribunals to hear investor–State dispute resolution cases and to promote trade liberalisation that will enable their products to dominate emerging markets [23]. They also create organisations to act as fronts through which they can influence the policy agenda.

Although transnational corporations’ investment may improve wages and working conditions in some cases, it can also worsen them [24]. Large multinational corporations determine the working conditions of workers by either shifting jobs to countries with weaker labour protections or by threatening to do so, thereby reducing the power of collective bargaining and legislation on health, safety and minimum wages [25]. They may use a complex web of deregulated global finance, for example, to transfer large payments and internal loans that shift their reported profits to low-tax jurisdictions to minimise what they have to contribute to the creation of both domestic and international public goods.

## 4. What Are Oral Health Inequalities?

Inequalities in health arise from the unequal distribution of power, income, goods and services, globally and nationally. Health inequalities are unfair, unjust and unacceptable [26]. Oral diseases disproportionately affect socially marginalized groups, with Beal suggesting that “it’s the poor wot gets the blame” [27]. These differences, which are apparent through all stages of the life course and across all countries, are called “oral health inequalities”. Oral health inequalities reflect differences in rates of poor oral health (conditions which are largely preventable) and access to timely, affordable and acceptable dental care. Examples include differences in clinical indicators of dental disease, including dental caries, periodontal disease and oral cancer, and self-perceived oral health from global ratings and assessments of oral health-related quality of life [28]. The literature suggests that oral health inequalities are caused by the broad conditions in which people are born, grow, live, work and age; the so-called “social determinants”. The literature suggests that the impacts of dental diseases are experienced more profoundly among socially vulnerable groups, including the elderly, the disabled, the incarcerated, ethnic minorities, those living in geographically remote locations, refugees and the socially displaced [27]. A multitude of population-level approaches to reducing oral health inequalities have been implemented across many countries over the years, with varying success. The most successful has been the use of fluoride in its various forms [29].

## 5. Industries That Directly Impact Oral Health Inequalities

The corporate determinants of health, which are included in the broad social conditions in which people live, have a profound and sustained impact on oral health inequalities [30,31]. The three most important dental diseases at the global level (dental caries, periodontal disease and oral cancer) are all heavily influenced by the products and practices of multinational corporations, particularly the sugar industry (dental caries), the alcohol industry (oral cancer, dental trauma) and the tobacco industry (periodontal disease, oral cancer) [31]. Large corporations contribute to oral health inequalities, primarily through their influence on the regulatory structures governing their activities; a key example is their shaping of the regulation of tobacco products in economically disadvantaged countries. Another is sugar, with Kearns and Watt demonstrating how the 40-year inertia of the World Health Organization in failing to endorse national sugar restrictions as a solution to dental caries was due to the sustained strategic actions of the World Sugar Research Organisation and the International Life Sciences Institute, both industry-funded groups with economic interests in sugar (and a history of influence that is only now beginning to be understood) [32]. These strategies included debating statistics, sponsoring conflicting studies and influencing committees. Emerging evidence on the impacts of taxation on sugar-sweetened beverages is encouraging [30], although there has been strong opposition from the sugar industry on this.

## 6. A Conceptual Framework of the Corporate Determinants of Health and Oral Health Inequalities

**Figure 1:**
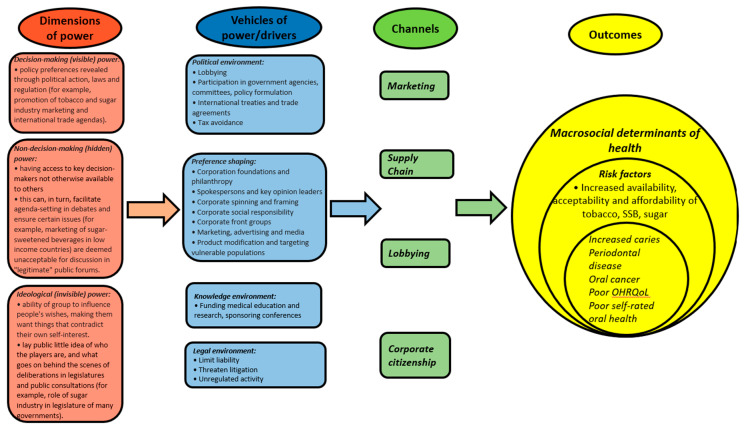
Conceptual framework of corporate determinants of health and oral health inequalities, based on Lukes’ three faces of power theory and Kickbusch’s processes through which transnational corporations exert influence.

“Ensuring healthy lives and promoting well-being for all ages” is the third goal in the United Nation’s Sustainable Development Goals set for 2030 [33]. Key performance indicators for achieving these goals include universal health care and prevention/treatment of non-communicable diseases [34]. Oral health clearly falls within this remit through, for example, reducing oral health inequalities through prevention and treatment at a population level. However, the main prevention strategies of the SDGs focus on the risks associated with poor diet, tobacco use and alcohol consumption; this is clearly an emphasis that is largely placed on lifestyles and personal responsibility. Such an approach ignores the limited control that many people have over their circumstances and their exposure to the marketing strategies of transnational corporations [35]. Based on Lukes’ three faces of power theory [12] and Kickbusch’s processes through which transnational corporations exerted influence [4], we constructed a framework through which the corporate determinants of health and oral health inequalities might be better conceptually understood (Figure 1). The purpose of this framework is to enable public health researchers, practitioners and policy makers to better understand the knock-on effects of the power that corporations exert, in all its guises, on the macrosocial determinants of health that include oral health outcomes [4]. The conceptual framework also indicates pathways by which the actions of corporations impact oral health inequalities, which could be empirically tested in future research.

## 7. How Can the Dental Profession Counter the Corporate Determinants of Oral Health?

As highlighted by Watt et al., dental professional organisations, institutions, peak bodies (an association of industries or groups with allied interests that act on behalf of all members when lobbying government or promoting the interests of the members) and policy makers need to apply greater pressure on the corporations that have the most impact on sustaining oral health inequalities (notably, the sugar, alcohol and tobacco industries) [31]. This includes refusal of funding, requests for transparency and greater scrutiny of perceived conflicts of interest when involved in any form of collaboration [22]. Public health advocates must become more skilled at recognising and understanding problem definition claims about sugar, alcohol, tobacco and dental diseases made by powerful vested interests and be prepared to counter them.

## 8. Conclusions

Actions the public health community can undertake to challenge the corporate determinants of oral health include demonstrating where and how people’s choices are structured by forces outside their immediate control, and asking whether it is acceptable that corporations whose products and actions clearly impact negatively on population oral health still retain a seat at health policy tables. We can align with other social movements committed to challenging the inequity of power in the hands of these corporations [36], and thus begin the process of holding powerful global corporations to account for their impacts on oral health.
